# Feasibility, acceptability, and outcome responsiveness of the SYMPERHEART intervention to support symptom perception in persons with heart failure and their informal caregivers: a feasibility quasi-experimental study

**DOI:** 10.1186/s40814-023-01390-3

**Published:** 2023-10-04

**Authors:** Gabrielle Cécile Santos, Maria Liljeroos, Kelly Tschann, Kris Denhaerynck, Justine Wicht, Corrine Y. Jurgens, Roger Hullin, Petra Schäfer-Keller

**Affiliations:** 1 School of Health Sciences, HES-SO University of Applied Sciences and Arts Western Switzerland, Fribourg, Switzerland; 2 Institute of Higher Education and Research in Healthcare-IUFRS, University of Lausanne, Lausanne University Hospital, Lausanne, Switzerland; 3 Department of Health, Medicine and Caring Sciences, Linköping University, Linköping, Sweden; 4 Centre for Clinical Research Sörmland, Uppsala University, Eskilstuna, Sweden; 5 Institute of Nursing Science, Department of Public Health, University of Basel, Basel, Switzerland; 6 Service d’Aide et de Soins à Domicile de La Sarine, Fribourg, Switzerland; 7 William F. Connell School of Nursing, Boston College, Chestnut Hill, Massachusetts USA; 8 Department of Cardiology, Lausanne University Hospital, Lausanne, Switzerland; 9 Faculty of Biology and Medicine, University of Lausanne, Lausanne, Switzerland

**Keywords:** Heart failure, Self-care, Symptom perception, Complex intervention, Informal caregivers, Feasibility, Acceptability, Clinical trial, Feasibility quasi-experimental study

## Abstract

**Background:**

Symptom perception is an important process of heart failure (HF) self-care that persons with HF need in order to master self-care management. It also leads to better patient outcomes. Symptom perception consists of body observation and analysis, which are both challenging. We aimed to test the feasibility, acceptability, and outcome responsiveness of a novel intervention (SYMPERHEART) delivered to persons with HF with their informal caregiver.

**Methods:**

We designed SYMPERHEART as a complex evidence-informed education and support intervention targeting body observation and analysis. We conducted a feasibility quasi-experimental study with a single group pre-post-test design. We included three subsamples: persons with HF receiving home-based care, their informal caregivers exposed to SYMPERHEART, and home-care nurses who delivered SYMPERHEART during 1 month. We assessed feasibility by recruitment time, time to deliver SYMPERHEART, eligibility rate, and intervention fidelity. We assessed acceptability by consent rate, retention rate, persons with HF engagement in body observation, and treatment acceptability. Outcome responsiveness was informed by patient-reported (PRO) and clinical outcomes: HF self-care and the informal caregivers’ contribution to HF self-care, perception of HF symptom burden, health status, caregivers’ burden, and HF events. We performed descriptive analyses for quantitative data and calculated Cohen’s d for PROs. A power analysis estimated the sample size for a future full-scale effectiveness study.

**Results:**

We included 18 persons with HF, 7 informal caregivers, and 9 nurses. Recruitment time was 112.6 h. The median time to deliver SYMPERHEART for each participant was 177.5 min. Eligibility rate was 55% in persons with HF. Intervention fidelity revealed that 16 persons with HF were exposed to body observation and analysis. Consent and retention rates in persons with HF were 37.5% and 100%, respectively. Participants engaged actively in symptom and weight monitoring. Treatment acceptability scores were high. Symptom perception and informal caregivers’ contribution to symptom perception were found to be responsive to SYMPERHEART. We estimate that a sample size of 50 persons with HF would be needed for a full-scale effectiveness study.

**Conclusions:**

SYMPERHEART was found to be feasible and acceptable. This feasibility study provides information for a subsequent effectiveness study.

**Trial registration:**

ISRCTN. ISRCTN18151041, retrospectively registered on 4 February 2021, ICTRP Search Portal.

**Supplementary Information:**

The online version contains supplementary material available at 10.1186/s40814-023-01390-3.

## Key messages regarding feasibility

### Uncertainties regarding feasibility

- Feasibility to recruit and include persons with HF and their informal caregivers in a home-based care setting for testing a complex evidence-informed intervention (SYMPERHEART) supporting symptom perception in HF
- Time needed to deliver SYMPERHEART and feasibility to deliver all intervention components to persons with HF
- Participant engagement in SYMPERHEART activities
- Acceptability to receive and deliver SYMPERHEART
- Outcome responsiveness of SYMPERHEART in persons with HF and their informal caregivers

### Key findings

- Eligibility rate in persons with HF and their informal caregivers was 55% and 100%, respectively.
- Consent rate in persons with HF and their informal caregivers was 37.5% and 63.6%, respectively.
- 177.5 min were needed to deliver SYMPERHEART for each person with HF across three meetings.
- 16/18 persons with HF were exposed to both body observation and body analysis.
- Participants engaged in symptom and weight monitoring during the 30 days of the intervention.
- SYMPERHEART was deemed acceptable by the persons with HF, their informal caregivers, and the nurses who delivered the intervention.
- Symptom perception by persons with HF and the informal caregivers’ contribution to symptom perception were both found to be responsive to SYMPERHEART.

### Implication of the feasibility findings for the design of the main study

- Results help to define challenges in identifying and recruiting persons with HF.
- Enhanced strategies to support intervention fidelity to attain full intervention delivery to all persons with HF should be considered, especially for body analysis using guided reflection.
- Based on the mean difference in the symptom perception variable, a sample size of 50 persons with HF is needed for a future randomized controlled trial.

## Background

Heart failure (HF) is associated with poor patient outcomes such as high morbidity and mortality and poor quality of life [1–3]. HF management is a priority and includes HF self-care in multidisciplinary programs [3]. Self-care is a naturalistic decision-making process whereby individuals perform behaviors to maintain health and respond to symptoms when they occur [4]. The authors of the Theory of Self-care of Chronic Illness recently integrated symptoms into the theory and recommended symptom interpretation and response to be part of self-care support [5]. Self-care management, i.e., response to symptoms when they occur, is associated with better patient outcomes such as better quality of life and improved event-free survival [3, 6, 7]. Importantly, symptom perception is initially required to attain adequate self-care management [8] and leads to better health [9].

Symptom perception consists of behaviors of body observation and analysis [10]. More specifically, “body observation” consists of body listening and symptom monitoring. “Body analysis” consists of recognition, interpretation, and labeling of symptoms [8]. Such behaviors may be challenging for persons with HF [10] and are often not optimally performed [10, 11].

The effect of multi-component interventions including HF self-care education has been evaluated [3]. Previous intervention studies supporting HF symptom perception [9, 12, 13] resulted in clinically relevant improvements in HF self-care maintenance, confidence [13], and management [12], as well as decreased symptom distress and number of symptoms [9, 14, 15]. Also, symptom perception behaviors were associated with improved HF self-care and health status, as well as decreased use of health care [9]. The impact of specifically targeting symptom perception needs to be further studied [16]. Home visits by nurses within multidisciplinary follow-up of chronic HF were reported to reduce mortality and HF hospitalization [3, 17]. Informal caregivers (i.e., family members and friends) [18] may play a key role in monitoring HF symptoms [19]. They may improve self-care in elderly, frail, and cognitively impaired persons with HF [20]. Although involving informal caregivers in HF self-care has been recommended [21, 22], research on their involvement in symptom perception is scarce.

We developed a complex intervention (SYMPERHEART) to support symptom perception in persons with HF combining body observation and body analysis and including informal caregivers. According to the Medical Research Council’s (MRC) framework for developing and evaluating complex interventions in health [23, 24], uncertainties may remain regarding the feasibility, acceptability, and outcome responsiveness. These uncertainties concern intervention delivery, acceptability, and magnitude of change, as well as procedures regarding participant eligibility and recruitment, which need to be addressed before intervention effectiveness can be evaluated [23, 24].

## Methods

### Aim

This study’s primary aim was to test the feasibility and acceptability of the SYMPERHEART intervention delivered by home-care nurses to home-dwelling persons with HF and their informal caregivers. The secondary aim was to test outcome responsiveness in persons with HF and their informal caregivers. For the former: HF self-care, perception of HF symptom burden, health status, and clinical outcomes. For the latter: the informal caregivers’ contribution to HF self-care and the caregivers’ burden.

### Study design

The design was a feasibility quasi-experimental pre-post-test study with measurements at baseline, post-intervention (1 month), and follow-up (3 months). A single group composed of persons with HF and their informal caregivers was exposed to SYMPERHEART. The study reporting is in line with the CONSORT extension for Pilot and Feasibility Trials Checklist [25], as reported in Additional file 1.

### Setting and sample

The study was conducted in a home-based service providing primary care to more than 2200 persons with any type of disease in a rural region of Western Switzerland, including a regional town. Three interlocking convenience samples composed the study sample. We targeted (a) 15 to 30 adults with confirmed HF, in New York Heart Association (NYHA) functional classes II–IV, receiving home-based care and living at home; (b) their informal adult caregiver who has at least one weekly contact; and (c) home-care nurses working in the study setting and trained to deliver SYMPERHEART [16]. The target sample size of 15 to 30 persons with HF was considered appropriate for a feasibility study [25–30] to inform intervention feasibility and acceptability, which was the study’s primary aim.

### SYMPERHEART intervention

SYMPERHEART is a complex evidence-informed education and support intervention [9, 10] aiming to support symptom perception in persons with HF. It is composed of components to support both body observation and body analysis and includes informal caregivers. The intervention, delivered through three meetings by home-care nurses, is detailed in Table 1.

**Table 1:** SYMPERHEART intervention

TIDieR items [8]	Intervention description
1. Brief name	SYMPERHEART supports SYMptom PERception in persons with HEART failure including their informal caregivers
2. Why, intervention rationale, theory, or goal	SYMPERHEART is a complex evidence-based intervention targeting both body observation and body analysis to support and educate persons with HF and their informal caregivers to monitor, recognize and interpret their HF symptoms, in order to guide symptom response Based on the situation-specific theory of HF self-care [8], symptom perception is a needed step to attain self-care management in the process of HF self-care. Based on evidence synthesis on symptom perception in HF [9, 10] and on care needs [31], the SYMPERHEART intervention was detailed [16] to be tested in the local setting
3. What, materials	Materials used to prepare the intervention delivery: • Patient-reported outcomes to identify HF self-care [32] and symptom burden [33] in persons with HF • Informal caregivers-reported outcomes to identify contribution to HF self-care [34] and caregiver burden [35] in informal caregivers of persons with HF Materials used to deliver the intervention to participants: • HF booklet of the Swiss Heart Foundation [36] • Paper graphs for daily symptom monitoring [13] • Digital weighing scale • Guided reflection questions [16] • Heartfailurematters.org web site Materials used to train the nurses delivering the intervention: • An intervention manual detailing how the intervention components are operationalized • A training manual detailing the learning objectives and the resources used during the teaching
4. What, procedures, and support activities	The SYMPERHEART intervention is composed of three intervention components to be delivered to persons with HF and their informal caregivers [16]: 1. **Intervention prerequisite**: the nurse identifies symptom perception barriers and facilitators [10], identifies HF self-care behaviors and individual symptom clusters based on patient-reported outcomes, discusses with the person with HF’s their main concerns related to HF, and supports self-care maintenance in discussion with the person using the HF booklet [36]. The nurse asks the informal caregiver their role related to the person’s HF and their wished role related to symptom perception, informs the informal caregiver about how symptom perception can be supported and about the heartfailurematters’ web site 2.** Body observation**: the nurse discusses individual symptom clusters with the person with HF in identifying the three most severe symptoms for daily self-monitoring. Persons with HF and informal caregivers are instructed on symptom monitoring with paper graphs and on weight and edema monitoring [13]. The nurse discusses symptom monitoring behaviors to facilitate symptom monitoring embedded in daily routine. A digital weighing scale is provided if needed. Weight gain or loss are discussed. Symptom response is guided with information on how to respond to symptoms in case of alarm signs [36] 3.** Body analysis**: the nurse uses person recall to support situation awareness about HF symptoms. Then, guided reflection questions are used both in persons with HF and their informal caregivers to support symptom recognition and interpretation [16]. The nurse informs the participants on self-care management activities [3]
5. Who provided	Home-care nurses who were previously trained by GCS with a one-day course on SYMPERHEART intervention components. All were registered nurses. The median years of professional experience was 10 (IQR 10) and the median years of professional experience in home-based care was 3 (IQR 5) Seven home-care nurses were trained to deliver the intervention, and five of them delivered the intervention. One nurse on maternity leave did not deliver the intervention and was replaced by GCS who delivered the intervention to one participant
6. How, modes of delivery	Face to face contacts with the person with HF with or without any informal caregiver
7. Where, location	Home visits at person’s with HF home
8. When and how much	The intervention was composed by three one-hour meetings delivered during a one-month period
9. Intervention tailoring	The intervention was tailored to the person’s self-care behaviors and individual symptom clusters, to the person with HF’s main concerns related to HF. The intervention was tailored to the informal caregiver's contribution in HF self-care, to the informal caregiver’s wished role in symptom perception and considering also caregiver burden
10. Modifications	None
11. How well intervention fidelity was planned, strategies used	Nurses filled an intervention fidelity checklist after each meeting. Monitoring intervention fidelity was done with one or several observations by GCS with each nurse at person’s with HF home filling the same intervention fidelity checklist Several strategies were used to support intervention fidelity and included the detailed description of the intervention components in an intervention manual available on French for the nurses, training the nurses to deliver the intervention, monthly team meetings with the nurses to maintain intervention fidelity, monitoring intervention fidelity during intervention delivery with each nurse, providing feedback about intervention fidelity to the nurses, and fidelity optimization by GCS enhancing fidelity during the monitoring of intervention fidelity
12. How well intervention fidelity was actual	17/18 persons with HF exposed to the intervention. 5/7 informal caregivers exposed to the intervention **1. Intervention prerequisite**: 15/18 persons with HF exposed to the total of activities, 2/18 exposed to several activities; 3/7 informal caregivers exposed to the total of activities, 2/7 exposed to several activities **2. Body observation**: 16/18 persons with HF exposed to the total of activities, 1/18 exposed to several activities; 5/7 informal caregivers exposed to the total of activities **3. Body analysis**: 14/18 persons with HF exposed to the total of activities, 2/18 exposed to several activities; 5/7 informal caregivers exposed to the total of activities

### Progression criteria

The progression criteria were defined as outcomes of feasibility and acceptability as described below. There were no predefined thresholds. We assessed progression success in monthly meetings with home-based care nurses delivering the intervention to judge the quality and progress of the intervention delivery. Thus, we used their inputs based on their practical experience to continually update our knowledge about the intervention feasibility and acceptability.

Feasibility was defined as success in delivering the intervention and in executing the procedures as planned. Acceptability was defined as the suitability of the intervention and the procedures from the perspective of the various participants including the intervention providers [37]. Outcome responsiveness, i.e., patient-reported outcomes (PRO) and clinical outcomes, were the secondary outcomes of the study.

### Feasibility

Feasibility was assessed by recruitment time, the time needed to deliver the intervention, eligibility rate, and intervention fidelity [38, 39]. Recruitment time included identifying potential persons with HF, verifying the HF diagnosis with the general practitioner if the diagnosis was not documented, assessing the eligibility of persons with confirmed HF, providing the study leaflet, identifying potential informal caregivers, giving information about the study, and collecting written informed consent. Intervention fidelity was measured by self-report checklists of the activities performed for each intervention component, as completed by the nurses after each intervention delivery. The intervention delivery was completed by a member of our team (GCS), who observed at least one intervention delivery with each nurse. We defined maximal fidelity as the number of persons exposed to all activities of each intervention component. Partial fidelity indicated the number of persons exposed to several but not all of the activities of the intervention components. We supported intervention fidelity via an intervention manual, sharing nursing notes on intervention components, and we trained and supported the nurses to deliver the intervention as per protocol [40].

### Acceptability

Acceptability was assessed by consent rate, retention rate, number of persons with HF engaging in body observation, and treatment acceptability among persons with HF, informal caregivers, and nurses [41]. The retention rate was calculated by comparing the number of participants retained during the 3-month study period and the number of lost to follow-up, separately for persons with HF and informal caregivers. Lost to follow-up was defined by unavailability of both PRO and clinical outcomes, or withdrawal from the study. Engagement in SYMPERHEART by persons with HF and informal caregivers was measured by the frequency of engagement in symptom and daily weight monitoring, based on paper graph documentation; as well as the number of responses to weight gain or weight loss of more than 2 kg in 1 to 3 days as documented on the paper graph and completed by nursing notes. Treatment acceptability was measured with the Treatment Acceptability and Preferences (TAP) measure [42, 43] adapted for this study. The TAP measure is composed of four treatment acceptability attributes: appropriateness, suitability, effectiveness, and willingness to comply [42]. The TAP measure has been previously used in French and German (for Switzerland) versions in our context in an 8-item adapted version [44]. For this study, three items [intervention coherence; participants’ confidence to perform body observation and body analysis from the HF Self-care Confidence scale [32] and the Caregiver Self-efficacy in Contribution to Self-care scale] were added to the 8-item adapted version, with a view to including acceptability components [45]. The resulting 11-item scale was slightly modified for wording according to its use in persons with HF, their informal caregivers, or the nurses who delivered the intervention. Additionally, we added an empty field at the end of the scale to allow participants to add comments.

### Outcome responsiveness

#### Outcomes in persons with HF

PRO in persons with HF included HF self-care, symptom perception confidence, and perception of HF symptom burden, as well as health status. HF self-care was measured using the Self-Care of HF Index (SCHFI) v.7.2, a 29-item three-scale instrument to measure self-care maintenance, symptom perception, and self-care management. SCHFI demonstrated adequate construct validity and good internal consistency in a sample of 631 adults with HF (global reliability index for multidimensional scales, respectively 0.75, 0.85, 0.70) [32]. The cutoff indicating adequate self-care is ≥ 70 for each scale. Half a standard deviation (SD) or an 8-point increase is defined as the minimally important change in the scores [46]. In addition, symptom perception confidence was measured with two items belonging to the self-care confidence scale [32].

Perception of HF symptom burden was measured with the Heart Failure Somatic Perception Scale (HFSPS) v.3, an 18-item instrument measuring symptom perception burden in persons with HF, across four subscales labeled dyspnea (6 items), chest discomfort (2 items), early and subtle (7 items), and edema (3 items) [33]. A 6-point Likert scale ranging from (0 = I did not have this symptom; 1 = not at all bothersome to 5 = extremely bothersome) assesses responses of persons with regard to the extent of having been bothered during the past week by HF physical symptoms [33]. The instrument has previously demonstrated adequate internal consistency (Cronbach’s alpha 0.90) as well as convergent and divergent validity in a sample of 378 persons with chronic HF [33].

Health status was measured with the Kansas City Cardiomyopathy Questionnaire (KCCQ) 12-item version [47, 48] that measures HF-related health status through physical limitation (3 items), symptom frequency (4 items), quality of life (2 items), and social limitations (3 items) [48]. Fair to good health status is described by KCCQ total scores from 50 to 74, and a score ≥ 75 indicates good to excellent health. A KCCQ 5-point score increase between baseline and follow-up indicates a clinical significant improvement, while a 3-point decrease indicates clinical significant deterioration [48]. The instrument has demonstrated good construct validity and test–retest validity (Intraclass coefficient correlation 0.92), in a sample of 4168 adults with HF [48].

Clinical outcomes were measured by the number of HF hospitalizations (due to cardiac decompensation) and number of deaths occurring during the 3-month study period. An HF hospitalization resulting in death was counted as two events. The length of stay for HF hospitalization was measured in days. Any other hospitalization reason as well as length of stay was recorded. Patient health records from a home-based care setting were used to collect these events.

#### Outcomes in informal caregivers

Outcomes in informal caregivers included caregivers’ contribution to HF self-care, caregiver symptom perception self-efficacy, and caregivers’ burden.

Caregivers’ contribution to HF self-care was measured with the Caregiver Contribution to Self-care of HF Index version 2 (CC-SCHFI 2) [34] which is a three-scale instrument to measure caregivers’ contribution in self-care maintenance, symptom perception, and self-care management. The CC-SCHFI 2 mirrors the SCHFI v.7.2 and has demonstrated adequate construct validity and good internal consistency (global reliability index for multidimensional scales 0.79, 0.86, and 0.85, respectively) in a sample of 277 caregivers [34]. Two items of the Caregiver Self-efficacy in Contribution to Self-care scale specifically concerning symptom perception were used to measure caregiver symptom perception self-efficacy in our study.

Caregivers’ burden was measured with the Zarit Burden Interview [49], a 22-item instrument measuring perceived burden in caregivers [50], with good reliability and validity as reported in 124 caregivers of persons with HF, with good internal consistency (Cronbach’s alpha 0.92) [35]. The cutoff indicating high caregiver burden is ≥ 17 [35].

### Study procedures

Persons with HF were screened for eligibility and identified by a nurse coordinator and the home-care nurses of the study setting during all the study period. HF diagnosis was confirmed by the general practitioner or cardiologist, if needed. Then, home-care nurses provided brief information about the study to the persons with HF. If they consented to share their contact information with the research team, they were fully informed about the study by the research nurse. Persons with HF were included by GCS or KT after full eligibility assessment and after providing written informed consent. Following persons with HF enrollment, authors GCS or KT screened informal caregivers for eligibility, informed them about the study, and included them. GCS or KT collected baseline data at the participants’ home who then also asked about preferences to be contacted for follow-up data collection. Socio-demographic data was collected using a questionnaire and during a participant interview. Clinical data were retrieved from home-based care records. Safety monitoring was conducted during the study until follow-up at 3 months [16]. Data about depressive symptomatology was collected from self-reports of the persons with HF using the Patient Health Questionnaire-2 (PHQ) [51]. Data about frailty was collected from nurses delivering SYMPERHEART using the Clinical Frailty Score (CFS) [52].

### Analysis

We performed descriptive analysis on the feasibility and acceptability variables. Descriptive statistics, mean absolute change, and Cohen’s d effect sizes were calculated to describe outcome responsiveness. A power analysis on the symptom perception variable at alpha level 0.05 and beta level 0.80 was conducted to estimate the necessary sample size for a future randomized controlled trial. We used the Statistical Package for Social Science 23 (IBM SPSS Statistics) and SAS 9.4 (SAS Institute, Cary, NC).

### Ethical considerations

The study was approved by the Human Research Ethics Committee of the Canton of Vaud, Switzerland (ref. 2020–01820). All persons with HF and informal caregivers provided written informed consent before being included in the study.

## Results

### Sample description

**Fig. 1:**
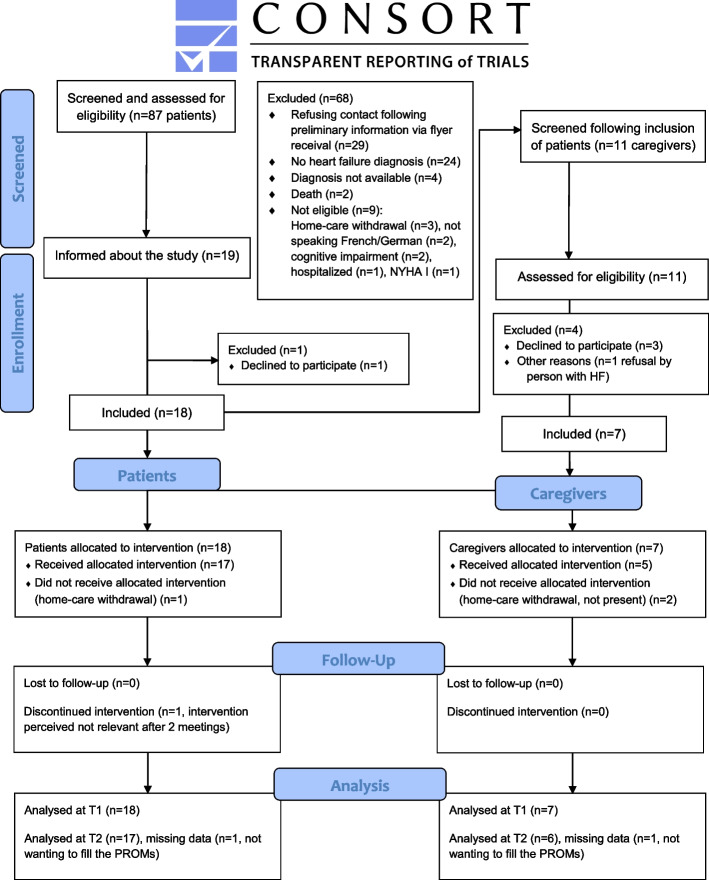
Study flow diagram with a design based on Eldridge et al. (2016) [25]

During the study period of 10 months, 87 persons and 11 informal caregivers were assessed for eligibility (see study flow diagram Fig. 1). Recruited to the study were 18 persons with HF, seven informal caregivers and nine nurses. Demographic and clinical characteristics at baseline are presented in Table 2. The majority (72%) of persons with HF were women, and 61% were in NYHA functional class II. Half of the sample of persons with HF had a clinical frailty score indicating frailty [52], with six persons mildly frail, two persons moderately frail, and one severely frail. Two persons had a patient-reported depressive symptomatology [51], and two persons with HF were cognitively impaired. The majority of informal caregivers (85%) were women. All informal caregivers were the spouse or a child of the person with HF, and the majority of whom (57%) were not living with the person with HF at the time. Nurses had a mean age of 38.1 years (± 13.0), and eight were female and one was male. Their median professional experience was 10 years (interquartile range, IQR = 10), and their median professional experience in home-based care was 3 years (IQR 5).

**Table 2:** Characteristics at baseline

	**Persons with HF (*n* = 18)** Mean ± SD or frequency (%)	**Informal caregivers (*n* = 7)** Mean ± SD or frequency (%)
**Age** (in years)	85.5 ± 7.2	64.7 ± 12.2
**Sex**		
Women	13 (72.2)	6 (85.7)
Men	5 (27.8)	1 (14.3)
**Education**		
Less than mandatory school	0 (0.0)	0 (0.0)
Mandatory school	11 (61.1)	2 (28.6)
Secondary education	4 (22.2)	3 (42.9)
Tertiary education	3 (16.7)	2 (28.6)
**Living situation**		
Living alone	11 (61.1)	0 (0.0)
Living with someone	7 (38.9)	7 (100.0)
**Received social support**^a^		
Yes	18 (100.0)	7 (100.0)
**Nature of relationship with the person with HF**	/	
Spouse		3 (42.9)
Child		4 (57.1)
**Nature of living situation**	/	
Living with the person with HF		3 (42.9)
Not living with the person		4 (57.1)
**Religion**		
Catholic	17 (94.4)	4 (57.1)
Protestant	1 (5.6)	1 (14.3)
Muslim	0 (0.0)	1 (14.3)
Other “no religion anymore”	0 (0.0)	1 (14.3)
**Race**		
Caucasian	18 (100.0)	7 (100.0)
**Time since HF diagnosis**		/
< 1 year	2 (11.1)	
1-4 year	3 (16.7)	
≥ 5 years	12 (66.7)	
Non-specified	1 (5.6)	
**NYHA**^b^** functional class**		/
NYHA II	11 (61.1)	
NYHA III	7 (38.9)	
NYHA IV	0 (0.0)	
**Previous HF hospitalization**		/
No	10 (55.6)	
Yes	8 (44.4)	
Yes, 1 hospitalization	7 (38.8)	
Yes, 3 hospitalizations	1 (5.6)	
**Comorbidities**		/
Cerebrovascular disease	6 (33.3)	
Renal disease	5 (27.8)	
Previous myocardial infarction	5 (27.8)	
Depressive symptomatology or anxiety^c^	5 (27.8)	
Cognitive impairment^c^	5 (27.8)	
Cancer, solid tumor	2 (11.1)	
Diabetes	1 (5.6)	
Chronic pulmonary disease	0 (0)	
*Instruments*		
Charlson Comorbidity Index	6.7 ± 2.1	
Patient Health Questionnaire-2^d^	0.9 ± 1.2	
Clinical frailty scale^e^	4.5 ± 1.1	
**Weight scale**		/
Having a digital weight scale at home	11 (61.1)	
No digital weight scale at home	7 (38.9)	
**Symptom perception confidence**^f^		
Routinely monitor condition	3.8 ± 0.9	4.0 ± 1.0
Recognize changes in health	3.8 ± 0.9	3.4 ± 1.1

^a^Assessed with the question “Do you have someone available you can count on?”

^b^NYHA New York Heart Association functional class

^c^Any note in medical or healthcare record

^d^PHQ-2 score ≥ 3 suggests clinically significant depression

^e^frailty if CFS > 4

^f^assessed with items 33 and 35 of the SCHFI 7.2, 1 = not confident, 3 = somewhat confident, 5 = extremely confident

### Feasibility

The time needed to screen, recruit, and enroll persons with HF and their informal caregivers was 112 h and 40 min. The median time needed to deliver the intervention was 60 min both at the first (IQR 12) and at the second meeting (IQR 8), and 55 min at the third meeting (IQR 15). The total median time to deliver the intervention to each participant was 177.5 min (IQR 45).

The eligibility rate was 55% in persons with HF, with 48 persons having been assessed as eligible among 87 potentially eligible persons. Of 11 informal caregivers assessed for eligibility, all were eligible, yielding a 100% eligibility rate among informal caregivers.

Intervention delivery fidelity as reported by the nurses and by GCS is presented in Table 3. There was a total of 50 intervention deliveries, with 17 deliveries for the first and the second meeting and 16 deliveries for the third meeting.

**Table 3:** Checklist fidelity components reported to be delivered or observed to be delivered at each meeting

	**First meeting**		**Second meeting**		**Third meeting**	
	Reported by nurse (*n* = 17)	Observed (*n* = 2)	Reported by nurse (*n* = 17)	Observed (*n* = 4)	Reported by nurse (*n* = 16)	Observed (*n* = 5)
**Intervention prerequisite (yes)**						
Symptom perception factors	15 (88%)	1 (50%)	6 (35%)	1 (25%)	6 (37%)	1 (20%)
Self-care behaviors	14 (82%)	1 (50%)	7 (41%)	2 (50%)	7 (43%)	1 (20%)
HF symptom burden	15 (88%)	1 (50%)	9 (52%)	1 (25%)	9 (56%)	1 (20%)
Persons’ with HF main concerns	15 (88%)	1 (50%)	5 (29%)	1 (25%)	4 (25%)	1 (20%)
Self-care maintenance support	14 (82%)	1 (50%)	3 (17%)	3 (75%)	4 (25%)	2 (40%)
**Body observation (yes)**						
Symptom clusters	16 (94%)	1 (50%)	13 (76%)	4 (100%)	10 (62%)	4 (80%)
Monitoring graphs	14 (82%)	1 (50%)	11 (64%)	4 (100%)	8 (50%)	3 (60%)
Self-care management	10 (58%)	1 (50%)	14 (82%)	4 (100%)	11 (68%)	3 (60%)
Alert symptoms and response	10 (58%)	1 (50%)	11 (64%)	4 (100%)	8 (50%)	3 (60%)
**Body analysis (yes)**						
Remembering symptoms	8 (47%)	1 (50%)	10 (58%)	3 (75%)	13 (81%)	4 (80%)
Guided reflection	4 (23%)	0 (0%)	13 (76%)	2 (50%)	15 (93%)	4 (80%)
**Informal caregiver (yes)**						
Informal caregiver present	4 (23%)	1 (50%)	5 (29%)	3 (75%)	6 (37%)	1 (20%)
Informal caregiver wished role	5	1	2	1	1	0
Heart failure matters information	3	0	1	0	2	0
Body observation implication	3	0	4	2	6	1
Body analysis implication	2	0	5	2	5	1

Table 3 illustrates the frequency of intervention component delivered with the percentage that it represents considering the number of participants exposed to the first, second, and third meeting

**Fig. 2:**
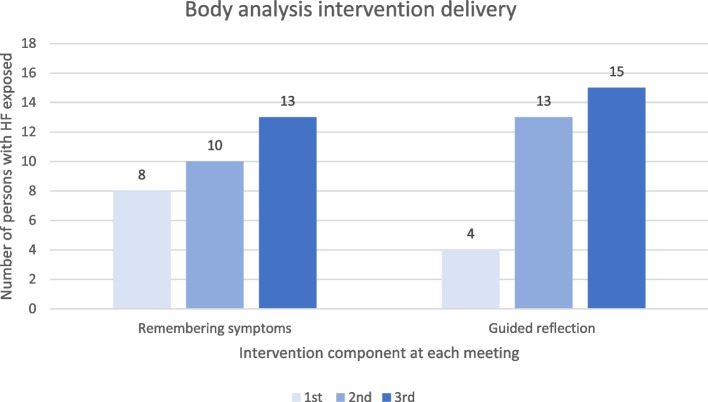
Self-reported nurse fidelity for body analysis

One person with HF was not exposed to the intervention, and another person with HF was exposed to only two meetings, respectively due to an intervention-independent reason of receiving no home-care anymore and due to a perception of having no benefit while continuing with the intervention. Over the three meetings, we noted a progression of the number of participants exposed to the three intervention components. First, the participants were exposed to the intervention prerequisite, then they were exposed to body observation and finally to body analysis. There was an increase in the number of persons exposed to body analysis intervention across meetings, i.e., those exposed to guided reflection were respectively four, 13 and then 15 persons over the first, second, and third meeting (Fig. 2).

Across all meetings, four, five, and six informal caregivers were present at the first, second, and third meeting (Table 3).

**Fig. 3:**
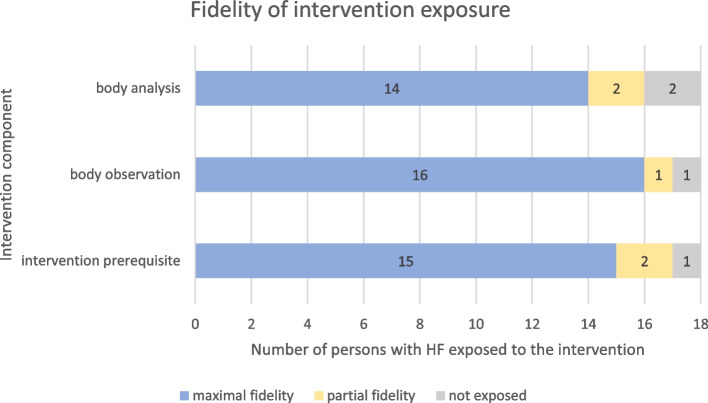
Fidelity of intervention exposure in persons with HF

Figure 3 provides an overview of intervention fidelity exposure in persons with HF. Sixteen persons with HF were exposed to the body observation and analysis components. In total, the three component interventions including all activities of intervention prerequisite, body observation, and body analysis were delivered fully to 13 persons. Four persons were exposed to several of the activities of the three component interventions. One person not exposed to the intervention received the intervention material at baseline, i.e., a weight scale and the booklet published by the Swiss Heart Foundation [36].

Five out of seven informal caregivers were exposed to the three intervention components, and two were not exposed to the intervention. Reasons for non-exposure were not receiving home-care anymore and poor health linked with fatigue.

### Acceptability

Of the 48 eligible persons with HF, 18 agreed to participate, yielding a 37.5% consent rate in persons with HF. Of the 29 persons who refused to be informed after having received initial study information, 17 did not provide a reason for refusal. Of those who did (*n* = 12), reasons included perception of a stable health condition and no perceived benefit (*n* = 4), symptoms of fatigue or feeling nervous (*n* = 2), the perceived burden of participating in additional healthcare activities (*n* = 3), caregiver burden (*n* = 1), not having enough time (*n* = 1), and considering it normal to have dyspnea as part of the natural aging process (*n* = 1). One person agreed to receive full study information, but then refused to participate mentioning not being available for participation.

Of the 18 persons with HF, 11 identified an informal caregiver for potential participation and seven did not. Reasons provided were not wanting to include an informal caregiver who was already busy (*n* = 4), not being available to participate (*n* = 1), and not wanting to disturb their informal caregiver (*n* = 1).

Of the 11 eligible informal caregivers who received study information, seven accepted, and four refused, yielding a 63.6% consent rate. Two persons provided reasons for non-participation, including not wanting to disturb the freedom of their family member (*n* = 1) and no interest in reading the information forms or filling out the questionnaires (*n* = 1).

Retention rate was 100% in persons with HF and informal caregivers, with none lost to follow-up. There was however missing data in the PRO follow-up questionnaires for two persons, due to non-interest; and there was one person who discontinued the intervention after two meetings, due to perceived non-relevance, but who did not ask to be withdrawn from the study.

Table 4 presents intervention acceptability for persons with HF, informal caregivers, and nurses. Overall, mean acceptability scores ranged from 3.5 and 4.8 across the three subsamples. In persons with HF, the highest acceptability was for feeling at ease, no opportunity costs, and a low burden to participate. The lowest acceptability was for intervention coherence and confidence to monitor one’s condition routinely. In informal caregivers, the highest scores were for the intervention corresponding to what is important, feeling at ease, low burden, and a perception that the intervention has a beneficial impact to live daily with the disease. For nurses, the highest scores were for the intervention corresponding to what is important, and no opportunity costs, and the lowest scores on confidence to monitor one’s condition and to recognize changes in health. Some persons with HF further commented on their responses to the adapted TAP measure, reported in the Additional file 2.

**Table 4:** SYMPERHEART intervention acceptability based on reported acceptability by the participants

**Acceptability component**	**Item extract**	**Persons with HF (*n* = 15–17)** Mean ± SD	**Informal caregivers** **(*n* = 4–6)** Mean ± SD	**Nurses (*n* = 5)** Mean ± SD
**Affective attitude** How an individual feels about the intervention	Appropriated in the current situation	3.8 ± 1.0	4.2 ± 0.4	4.0 ± 0.7
	Feeling at ease	4.5 ± 0.5	4.4 ± 0.5	4.2 ± 0.4
	Satisfied	4.1 ± 1.0	4.2 ± 0.4	3.8 ± 0.4
**Burden** The perceived amount of effort that is required to participate in the intervention	Participate again	3.8 ± 1.1	4.0 ± 0.7	4.6 ± 0.5
	Low burden to participate	4.2 ± 0.9	4.4 ± 0.5	3.8 ± 0.4
**Ethically** The extent to which the intervention has good fit with an individual’s value system	Corresponding to what is important, to values	3.7 ± 1.2	4.6 ± 0.5	4.8 ± 0.4
**Intervention coherence** The extent to which the participant understands the intervention and how it works	Coherent to monitor, recognize, and interpret symptoms	3.5 ± 0.8	4.0 ± 0.0	4.4 ± 0.5
**Opportunity costs** The extent to which benefits, profits or values must be given up to engage in the intervention	No opportunity costs	4.3 ± 0.8	4.5 ± 0.5	4.8 ± 0.4
**Perceived effectiveness** The extent to which the intervention is perceived as likely to achieve its purpose	Beneficial to live daily with the disease	3.7 ± 1.1	4.4 ± 0.5	4.0 ± 1.0
**Self-efficacy** The participant’s confidence that they can perform the behaviour(s) required to participate in the intervention	Confident to monitor condition routinely	3.6 ± 0.7	4.1 ± 0.9	3.4 ± 0.5
	Confident to recognize changes in health if they occur	3.7 ± 0.9	4.3 ± 0.5	3.4 ± 0.5

Sekhon et al. 2017, p.8 (adapted). Responses: 1 = completely disagree; 2 = disagree; 3 = neutral; 4 = agree; 5 = completely agree. Responses for self-efficacy based on SCHFI 7.2: 1 = not confident; 3 = somewhat confident; 5 = extremely confident. The scores should be rescaled from 0 to 4 if the results are compared with the original TAP [42]

Table 5 depicts participants’ responsiveness in the SYMPERHEART monitoring activities. Fifteen persons with HF monitored their dyspnea with a mean frequency of 22 times during the 30 days of intervention. Among persons weighing themselves, there were two situations of weight gain of more than 2 kg in 1 to 3 days documented on the monitoring graphs. Two situations of contacting the general practitioners by the persons with HF were documented, one related to weight gain with zero days of delay and one related to palpitation. Participants’ responsiveness in SYMPERHEART remained unknown for three persons with HF. Among those, one person was not exposed to the intervention and did not receive the symptom monitoring graph, and two persons did not send back the monitoring graphs. However, nursing notes suggest that both these persons sometimes used the monitoring graphs.

**Table 5:** Engagement in symptom and weight monitoring

	Number of persons with HF having monitored their symptom during 30 days, *n* (%)	Frequency of symptom monitoring during 30 days Mean ± SD
Monitoring dyspnea	15 (83)	22.2 ± 6.8
Monitoring fatigue	14 (77)	23.1 ± 6.2
Monitoring 3rd symptom	10 (55)	19.4 ± 9.2
Monitoring 4th symptom	5 (27)	23.2 ± 2.9
Monitoring weight	14 (77)	16.7 ± 11.8
Monitoring edema	14 (77)	16.4 ± 10.6

### Outcome responsiveness in persons with HF and their informal caregivers

HF self-care, health status, and symptom burden in persons with HF, as well as informal caregivers’ contribution to HF self-care and caregiver burden, are described in Table 6.

**Table 6:** Patient and caregivers-reported outcomes before and after the intervention exposure

	**Persons with HF (*n* = 17–18)**	**Informal caregivers (*n* = 6–7)**
	Mean ± SD	Mean ± SD
**Self-care/contribution to**		
Self-care maintenance		
Baseline	62.5 ± 12.2	60.0 ± 32.4
Post-intervention (30 days)	62.1 ± 18.1	63.5 ± 25.4
Follow-up (90 days)	58.2 ± 13.8	61.5 ± 18.1
Symptom perception		
Baseline	52.0 ± 17.7	58.9 ± 24.9
Post-intervention (30 days)	55.5 ± 17.0	69.1 ± 22.6
Follow-up (90 days)	59.8 ± 16.8	63.5 ± 18.8
Self-care management		
Baseline	38.6 ± 15.9	49.3 ± 25.6
Post-intervention (30 days)	39.2 ± 15.4	52.5 ± 20.9
Follow-up (90 days)	41.1 ± 19.9	51.5 ± 25.6
**Health status**, overall		/
Baseline	70.2 ± 15.6	
Post-intervention (30 days)	68.8 ± 14.1	
Follow-up (90 days)	70.1 ± 21.4	
Physical limitation		/
Baseline	66.6 ± 17.4	
Post-intervention (30 days)	64.3 ± 19.9	
Follow-up (90 days)	72.7 ± 28.4	
Symptom frequency		/
Baseline	77.4 ± 18.0	
Post-intervention (30 days)	75.6 ± 14.3	
Follow-up (90 days)	78.4 ± 22.5	
Quality of life		/
Baseline	65.9 ± 20.0	
Post-intervention (30 days)	63.8 ± 20.5	
Follow-up (90 days)	65.4 ± 20.5	
Social limitation		/
Baseline	69.7 ± 25.7	
Post-intervention (30 days)	70.0 ± 26.8	
Follow-up (90 days)	59.8 ± 31.6	
**Symptom burden**		/
Dyspnea		
Baseline	0.58 ± 0.54	
Post-intervention (30 days)	0.57 ± 0.71	
Follow-up (90 days)	0.79 ± 1.08	
Chest discomfort		/
Baseline	0.86 ± 1.09	
Post-intervention (30 days)	0.61 ± 0.79	
Follow-up (90 days)	0.94 ± 1.19	
Early subtle		/
Baseline	1.31 ± 0.74	
Post-intervention (30 days)	1.17 ± 0.64	
Follow-up (90 days)	1.16 ± 0.81	
Edema		/
Baseline	0.94 ± 0.96	
Post-intervention (30 days)	0.81 ± 0.90	
Follow-up (90 days)	1.11 ± 1.02	
**Caregiver burden**	/	
Baseline		18.5 ± 10.0
Post-intervention (30 days)		20.5 ± 15.1
Follow-up (90 days)		20.1 ± 6.2

*Note*. Higher scores indicate better self-care, health status, and higher symptom burden and caregiver burden. The cutoff indicating adequate self-care on the SCHFI and CC-SCHFI is ≥ 70 for each subscale. Fair to good health status is described by KCCQ scores from 50 to 74, and scores ≥ 75 indicate good to excellent health. Scores of the HFSPS are 0 = not had this symptom, 1 = not at all bothersome, 5 = extremely bothersome. The cut off indicating caregiver burden on the Zarit Burden Interview is ≥ 17 considered as high burden

#### HF self-care

All self-care scores were too low (i.e., < 70) at all measurement points (Table 6). However, symptom perception increased post-intervention and further increased at follow-up in persons with HF, by 7.6 points compared to baseline. Similarly, caregiver contribution to symptom perception increased by 10.2 points from baseline to post-intervention (T1) and decreased thereafter by 2 points. Caregiver contribution to symptom perception was 69.1 post-intervention and was the highest self-care score.

#### Health status

Health status in persons with HF was similar in all dimensions and across measurement points, except for the physical dimension which increased by 5 points at follow-up and the social limitation dimension that had an 8-point decrease at follow-up (Table 6).

#### Symptom burden

Persons with HF had a median of seven symptoms at baseline (min 2, max 17, Q1 3.75, Q3 8.00). We found the highest scores for symptom burden in persons with HF for early subtle symptoms with fatigue and nocturia, all of which were classified as the most bothersome compared with other physical symptoms (Table 6).

#### Caregiver burden

We found caregiver burden scores reaching the cut-off score for high caregiver burden at all measurement points (Table 6).

#### Mean absolute change in outcomes for persons with HF and informal caregivers

Mean absolute change in HF self-care, caregiver contribution to HF self-care, health status, symptom burden, and caregiver burden are presented in Table 7. For symptom perception, medium effect sizes were observed in both samples at follow-up, and a large effect size was observed in caregiver contribution to symptom perception post-intervention, all in favor of a clinical increase in HF symptom perception at all times of measurement as compared to baseline. A large effect size was also observed among informal caregivers, in favor of a clinical increase in caregiver contribution to self-care management between baseline and follow-up (Table 7).

**Table 7:** Mean absolute change between pre and post intervention and effect sizes

	Persons with HF (*n* = 13–18)	Informal caregivers (*n* = 6–7)
	Mean ± SD/effect size	Mean ± SD/effect size
**HF self-care**		
Self-care maintenance		
Baseline to post-intervention (+ 30 days)	− 0.3 ± 16.1 / − 0.01	3.5 ± 12.4 / **0.28**^a^
Baseline to follow-up (+ 90 days)	− 5.9 ± 11.7 / − **0.50**^b^	7.8 ± 33.5 / **0.23**^a^
Symptom perception		
Baseline to post-intervention (+ 30 days)	3.4 ± 15.4 / **0.22**^a^	10.2 ± 7.4 / **1.37**^c^
Baseline to follow-up (+ 90 days)	7.6 ± 13.6 / **0.55**^b^	8.2 ± 16.3 / **0.50**^b^
Self-care management		
Baseline to post-intervention (+ 30 days)	0.6 ± 16.9 / 0.03	3.1 ± 17.6 / 0.17
Baseline to follow-up (+ 90 days)	2.9 ± 18.6 / 0.15	8.0 ± 9.1 / **0.87**^c^
**Health status**, overall		
Baseline to post-intervention (+ 30 days)	− 1.3 ± 9.3 / − 0.13	
Baseline to follow-up (+ 90 days)	− 1.5 ± 14.2 / − 0.10	
Physical limitation		
Baseline to post-intervention (+ 30 days)	− 2.3 ± 13.4 / − 0.17	
Baseline to follow-up (+ 90 days)	5.0 ± 26.3 / 0.19	
Symptom frequency		
Baseline to post-intervention (+ 30 days)	− 1.7 ± 19.1 / − 0.08	
Baseline to follow-up (+ 90 days)	− 1.4 ± 22.2 / − 0.06	
Quality of life		
Baseline to post-intervention (+ 30 days)	− 2.0 ± 17.2 / − 0.11	
Baseline to follow-up (+ 90 days)	− 2.9 ± 15.6 / − 0.18	
Social limitation		
Baseline to post-intervention (+ 30 days)	1.7 ± 15.0 / 0.11	
Baseline to follow-up (+ 90 days)	− 8.3 ± 20.4 / − **0.40**^a^	
**Symptom burden**		
Dyspnea		
Baseline to post-intervention (+ 30 days)	− 0.00 ± 0.71 / − 0.00	
Baseline to follow-up (+ 90 days)	0.19 ± 1.00 / 0.19	
Chest discomfort		
Baseline to post-intervention (+ 30 days)	− 0.25 ± 1.46 / − 0.17	
Baseline to follow-up (+ 90 days)	0.11 ± 1.55 / 0.07	
Early subtle		
Baseline to post-intervention (+ 30 days)	− 0.14 ± 0.77 / − 0.18	
Baseline to follow-up (+ 90 days)	− 0.11 ± 0.89 / − 0.12	
Edema		
Baseline to post-intervention (+ 30 days)	− 0.12 ± 0.77 / − 0.15	
Baseline to follow-up (+ 90 days)	0.25 ± 0.76 / **0.32**^a^	
**Caregiver burden**		
Baseline to post-intervention (+ 30 days)		2.0 ± 13.5 / 0.14
Baseline to follow-up (+ 90 days)		4.0 ± 9.8 / **0.40**^a^

*Note*. Small, medium, and large effect sizes are annotated in bold in the table. Other effect sizes were found to be smaller than small effect size. The mean absolute change in this table can be different than the difference of the means that are reported in Table 6. This difference can be explained by missing data at follow-up in HF self-care measurement and by missing data in some of the items of health status measurement, as well as by the small sample size

^a^Small effect size ≥ 0.2

^b^medium effect size  ≥0.5

^c^large effect size ≥ 0.8

#### Sample size calculation

Assuming a mean difference of 7.8 in the symptom perception variable, a standard deviation of 17 and a correlation of 0.56 between paired measurements, a simulation was conducted to reach a power of 80% at a significance level of 0.05, indicating that a sample size of 50 would be needed for a future parallel randomized controlled trial.

#### Event

During the 90-day follow-up period, one hospitalization for cardiac reason occurred, with a length of stay of 21 days (6 days at hospital and then 15 days in rehabilitation).

#### Harms

No serious adverse events were identified that might have been related to the SYMPERHEART intervention or to the study procedures during the study period and the follow-up period, suggesting the intervention and the study procedures to be safe.

## Discussion

The results of this study provide information regarding procedural, methodological, and intervention uncertainties, as well as regarding acceptability and outcome responsiveness. Our results indicate that the intervention was both feasible and acceptable for this sample. Symptom perception was responsive to SYMPERHEART and participants engaged in monitoring activities. Testing the feasibility and acceptability of a complex intervention in local context is crucial to anticipate issues related to any further effectiveness studies [24, 53]. Because of the importance of recruitment [54], and because clinical trials may fail due to recruitment issues [23], we have investigated key elements regarding procedures, feasibility, and acceptability. The information acquired in this study will help to prepare recruitment for a future study with persons with HF in a similar setting.

### Uncertainties regarding procedures and methodology

This study’s eligibility rate was reduced because 24 persons did not have the formal diagnosis of HF and this diagnosis could not be confirmed for four additional persons. Based on the number of persons receiving home-based care in the study area, we had anticipated that we would identify about 150 persons living with an HF diagnosis. In practice, we identified less than a third of this number. Participant recruitment indicated how challenging the identification and recruitment of persons with HF was. A possible absence of positioning of the general practitioners about the HF diagnosis could indicate an underdiagnosed HF population. This converges with the challenge to identify persons with HF in general practice [55]. In the context of a lack of specialized and dedicated roles for nurses for persons with HF, we argue that nurses need to have access to the HF diagnosis if they are meant to support HF self-care and symptom perception.

### Uncertainties regarding feasibility

The intervention components were delivered during three meetings with close to maximal fidelity. As we observed a progression in the number of exposures to the different intervention components over the three meetings, this finding suggests that three encounters will be needed to deliver the intervention as per protocol. Specifically, close to maximal fidelity was reached in (a) adherence to components as described in the protocol; (b) dose of intervention considering the amount (length of each interaction), the frequency of meetings delivered as planned in the one-month duration; (c) participant responsiveness considering the engagement of persons with HF in the intervention; and (d) program differentiation with intervention components that distinguish from other treatments or interventions [40]. Our results report on fidelity in a comprehensive manner, as we assessed fidelity at both theoretical and operational levels, with fidelity to delivered intervention components based on theory, on dose of delivery, and on participant engagement in the intervention [40]. Furthermore, we used various facilitation strategies with description of a replicable intervention in an intervention manual, training to deliver the intervention, monitoring intervention fidelity [40], and support to deliver the intervention as per protocol. During fidelity monitoring of intervention delivery, we observed that the use of guided reflection questions was often missing. Indeed, guided reflection questions are inherent to SYMPERHEART and were completed during meeting observation to maximize fidelity. Given symptom recognition and interpretation outcomes [9], given that intervention supporting symptom monitoring combined with symptom recognition and interpretation should be preferred to symptom monitoring support alone [9], and given the importance of learning based on previous experience for HF self-care [56, 57] and for adequate symptom perception [58], body analysis components are important to be delivered to support all the symptom perception process.

### Uncertainties regarding intervention acceptability

Our study’s consent rate was comparable to the HF SMART pilot RCT that tested a similar intervention [13], and lower than the UTILE pilot RCT conducted in the same region in Switzerland [44] both studies included inpatients. On contrast this study was conducted in a home-based care setting where nursing research is rarely carried out in the Swiss context.

This study’s high retention rate and high acceptability scores suggest high acceptability of both study procedures and intervention acceptability. Among the two persons with HF with lower acceptability scores, one described a burden related to visual and hearing difficulties and would not participate again if the study was reconducted, and the other was the person discontinuing the intervention.

Engagement in symptom and weight monitoring provide additional information about intervention fidelity. Results related to weight monitoring suggest that this behavior is suboptimal in persons with HF, similar to low weight monitoring levels worldwide. Many persons with HF have reported monitoring their weight never, rarely, or sometimes [11]. Weight monitoring is also inadequate in our local context [31]. However, it is possible that persons with HF monitored their weight more than 16 times during the 30-day intervention duration but did not report it each time on the monitoring graph, as data collection and interpretation were conducted in a conservative manner. The mean score for the single weight monitoring item on SCHFI informs about the patient-reported engagement in weight monitoring with an increase in this self-care behavior (mean score 3.4 at baseline and 4.3 post-intervention, standardized score 68 and 86, respectively, data not shown).

### Uncertainties regarding outcome responsiveness and intervention impact

#### Self-care and contribution to self-care

Our findings of a medium effect size in HF symptom perception at follow-up indicate HF symptom perception responsiveness with a clinically relevant change following the intervention. These findings are comparable to previous studies testing similar interventions with clinical improvements in HF self-care maintenance [13] and management [12]. Importantly, the impact of SYMPERHEART on HF symptom perception in this study concerns an elderly sample of persons with HF, frail, and multimorbid, with the intervention delivered in the environment of home-based care. Several HF self-care influencing factors including cognitive impairment, depression, and symptoms [4] all concern this sample of persons with HF as shown in participants’ characteristics, e.g., with a frailty score that is in line with the 44% prevalence of frailty in the HF population [59]. Persons with HF who are both frail and cognitively impaired have the poorest outcomes regarding mortality and HF hospitalizations [60]. This indicates the need to consider this high-risk population in further interventions.

Our findings of increases in symptom perception in persons with HF between post-intervention and follow-up suggest that learning and behavior change take time. These findings echo an individual patient data meta-analysis reporting that longer duration of HF self-management intervention is an intervention characteristic related to better patient outcomes [7]. In our study, some persons with HF may still have been exposed to the intervention after the end of the per protocol 30-day intervention period. This was due to home-care nurses integrating aspects of the intervention into usual care. Evidence for this was obtained by conducting qualitative interviews with the intervention nurses (data not shown). Also, it could have been that the involvement of informal caregivers reporting an important increase in symptom perception at post-intervention may have impacted symptom perception in persons with HF. In a recent randomized controlled study, informal caregivers were described to possibly potentiate the effect of intervention supporting HF self-care. There, self-care management in persons with HF was improved when informal caregivers were involved [61]. It may also be the case in our study that the involvement of informal caregivers increases the impact of the intervention.

Our results inform on improved mean symptom perception and self-care management in informal caregivers than in persons with HF at different times of measurement. In comparison with persons with HF, the higher responsiveness in caregivers’ contribution to HF self-care immediately after the intervention exposure might be explained by influencing factors [10]. In our study, informal caregivers were younger and might have been in better health than the persons with HF. Also, including informal caregivers might have increased their confidence to contribute to HF symptom perception.

#### Health status

The findings regarding the KCCQ mean overall score are in line with the scores of NYHA class II patients [48]. The improvement in physical limitation at follow-up is interesting. It might be related to better self-care scores in our sample, as an increase in self-care was previously found to influence perceived health status even when SCHFI scores were low (but above the 15th percentile) [46, 62]. Our observed stability in overall health status over time and improvement regarding physical limitation are in keeping with the treatment goals of persons with HF who wish not to get worse, to improve physical function and to decrease symptoms [63]. Regarding the findings on worsening social limitation, this could be related to the COVID-19 pandemic situation.

#### Symptom burden

The perception of symptom burden findings illustrate that the sample included mainly persons with HF in NYHA class II who are only slightly limited at physical activity. This fits in with our findings on symptom frequency of the KCCQ-12 domain with the best health status results. Importantly, higher age was associated with fewer HF symptoms in persons older than 74-years old [64] and older persons with HF were described to have more difficulty than younger persons to detect dyspnea [65]. Persons with HF frequently avoid physical activity in order to feel less burdened by HF symptom as dyspnea or fatigue [66, 67]. This may be the case also in this elderly and frail sample. Our findings indicate a lower symptom burden than what has been reported in HF outpatients who suffer from numerous symptoms [64]. However, persons with HF included in this study perceived a median number of seven symptoms and up to 17 symptoms of HF. Most of them monitored their dyspnea, fatigue; some of them monitored a third or a fourth symptom, indicating an opportunity for symptom perception despite claiming to be only slightly limited by symptoms. Regarding the elderly nature of our sample, it may be possible that most of these persons have difficulty detecting their HF symptoms, stressing the importance to support both symptom monitoring and symptom recognition with an individualized intervention in order to tend to adequate HF self-care. Supporting older persons to monitor their dyspnea, to recognize, and to interpret an increase in dyspnea is of utmost importance as patient perception of dyspnea can predict hospitalization [68] and even event-free survival [33].

### Uncertainties regarding the integration of caregivers in supporting symptom perception in HF

#### Caregiver burden

We found a high informal caregiver burden at all measurement points. Interestingly, less than half of informal caregivers were living with the person with HF, indicating that their involvement in supporting the person with HF and burden might be independent from whether they live with them or not. Similarly, previous studies reported the burden of informal caregivers of persons with HF, experiencing negative impact on physical and mental health [69, 70].

#### Integration of caregivers in the intervention

Our results show that less than half of persons with HF in our sample were able or willing to participate with an informal caregiver, as a dyad. This small proportion should be taken into account for future studies targeting either dyads or individuals with HF on their own. In addition to the seven informal caregivers participating in the study, three other informal caregivers participated in the intervention without being included as participants in the study. The exposure occurred because these informal caregivers were at the home of the person with HF when the intervention took place and expressed the wish to be present during intervention delivery. The inclusion criteria for the informal caregivers may seem relatively wide: only a weekly contact was required to be eligible. However, most persons with HF in the study area live alone, which is in keeping with general population statistics in Switzerland where one third of persons aged over 65 live alone, a proportion that increases with age [71]. Therefore, requiring daily contacts would have implied losing a significant proportion of potential study participants.

In our study, the caregiver burden score increased, suggesting a higher burden after intervention delivery with a small effect size at follow-up compared to baseline. Acceptability scores in informal caregivers nevertheless suggest that a low burden was associated with participating in the study. We noted an increase of 31 points in caregiver burden between baseline and post intervention in one informal caregiver, who explained that the person with HF has recently been transferred to a nursing home, due to a worsening health condition. The increase of burden in this situation can be attributed to the evolution of HF and comorbidities and to the HF trajectory which tends to worsen over time [72]. Still, the caregiver burden needs to be considered in future studies. Our results converge with international literature indicating no harm for patients nor informal caregivers to include informal caregivers in self-care support [19].

### Strengths and limitations

One strength of this study is the state-of-the-art testing of a complex intervention guided by the MRC’s framework for developing and evaluating complex interventions in pilot testing the intervention feasibility and acceptability [23, 24]. Reproducibility of the study is possible thanks to the description of detailed methods including intervention components, and thanks to the publication of the study protocol [16].

Based on their definition, feasibility, and acceptability could be assessed differently. In this study, several measures informed intervention feasibility and acceptability from the perspective of persons with HF, their informal caregivers, and nurses having delivered the intervention. To our knowledge, this study is the first to report HF symptom perception description in a Swiss sample of persons with HF. It is also the first to report caregiver contribution to HF self-care in Switzerland. Regarding intervention magnitude of change, a strength is the internal validity with the use of valid and reliable instruments to collect data on PRO and caregivers reported outcomes. Importantly, this study informed the intervention as feasible and acceptable in the working practices of nurses caring for elderly persons with HF.

Several limitations relate to the study design, the sampling method, and the sample size. Without using a control group, between group differences cannot be assessed. Also, this study did not provide information on intervention impact for different subgroups, e.g., regarding persons with HF phenotypes of reduced, mildly reduced, and preserved left ventricular fraction ejection, as well as for frail persons, or having different comorbidities. Furthermore, controlling for clinical variables was not foreseen in this study but should be included in the effectiveness study.

Without a national nor a regional registry of persons with HF, we were unable to have a random sample. Selection bias could have occurred. However, the sample has characteristics which are typical for a clinical sample of persons with HF living in the community and needing home-based care. External validity and generalization of the results are not possible given the study design and the small sample included. We initially planned a sample size of 30 persons with HF and 20–30 informal caregivers [16], a sample size which was beyond the size possible to recruit during the study period. Obviously, we overestimated the number of eligible persons with HF and we underestimated the barriers to identify them. This was possibly due to an underdiagnosis of HF, a relevant issue also outside our context [55]. Thus, this feasibility study prevented the failure of running a large study. Contamination bias is possible if persons with HF had had prior exposure to HF education by home-care nurses. We consider this unlikely as HF self-care support is not part of usual care in the study setting. We also might have optimized fidelity through intensifying direct supervision and enhancing adherence to protocol [38].

Finally, we did not define progression criteria thresholds. Our results could be used to help define progression criteria thresholds with a traffic light approach guiding the decision to stop, amend, or proceed to the future effectiveness study [73]. Indeed, recent recommendations on progression criteria should be considered [74].

### Implications for practice and research

SYMPERHEART is a promising intervention. Given the importance of symptom perception in the HF self-care process, and given symptom perception responsiveness both in persons with HF and in informal caregivers, a standardized intervention is recommended to support symptom perception. Given caregiver burden already being present at baseline, we suggest to monitor caregiver burden and to offer a response to a burden increase to protect the health of informal caregivers.

Further research is needed to evaluate the SYMPERHEART intervention effectiveness before implementing the intervention in clinical practice. We suggest to measure its impact on HF self-care, caregiver contribution to HF self-care, health status, and caregiver burden. Perception of symptom burden is useful to identify individual symptoms for monitoring and should be further used to deliver a personalized intervention. Furthermore, core elements of the new MRC’s framework for developing and evaluating complex interventions need to be addressed for future steps of the SYMPERHEART intervention evaluation and implementation. Involving key stakeholders [53], particularly persons with HF and their informal caregivers as collaborating in the research should be considered for the next steps.

## Conclusions

SYMPERHEART, a novel complex intervention, was deemed feasible and acceptable in the working practices of home-based care nurses. The impact of SYMPERHEART on HF symptom perception both in elderly persons with HF and their informal caregivers is noteworthy. By expanding the current understanding of HF symptom perception interventions and outcomes in persons with HF and their informal caregivers, this study contributes to the body of evidence on intervention development to enhance HF self-care in order to contribute to better patients and clinical outcomes. The future effectiveness study, a parallel randomized controlled trial evaluating the effectiveness of the SYMPERHEART intervention compared to usual care, needs a setting where 50 persons with HF can be reached.

### Supplementary Information

**Additional file 1.** CONSORT extension for Pilot and Feasibility Trials Checklist.

**Additional file 2. **Participants comments provided filling adapted TAP measure.

## Data Availability

The datasets generated and analyzed during this study are available from the corresponding author on reasonable request. It will concern individual de-identified participant data of those participants having accepted to share their data by signing an additional informed consent form.

## Abbreviations

CC-SCHFI 2: Caregiver Contribution to Self-care of Heart Failure Index version 2
CFS: Clinical Frailty Score
HF: Heart failure
HFSPS: Heart Failure Somatic Perception Scale
IQR: Interquartile range
KCCQ: Kansas City Cardiomyopathy Questionnaire
MRC: Medical Research Council
NYHA: New York Heart Association
PHQ: Patient Health Questionnaire
PRO: Patient-reported outcome
SCHFI: Self-Care of Heart Failure Index
SD: Standard deviation
SYMPERHEART: A novel complex intervention to support HF symptom perception
TAP: Treatment Acceptability and Preferences
