# Supplementary material for: Feasibility, acceptability, and outcome responsiveness of the SYMPERHEART intervention to support symptom perception in persons with heart failure and their informal caregivers: a feasibility quasi-experimental study
Source: Pilot Feasibility Stud. 2023 Oct 4;9:168. doi: 10.1186/s40814-023-01390-3 (PMC10548691; doi:10.1186/s40814-023-01390-3)
Supplement: Supplementary file 2 — Additional file 2. Participants comments provided filling adapted TAP measure. [file 40814_2023_1390_MOESM2_ESM.docx]

**Additional file 2.** Participants comments provided filling TAP measure

Several persons with HF commented their TAP answers. Notes included one person to be satisfied, one appreciated the interactions with the nurse and said to pay more attention to symptom monitoring, one reported to be more aware of his/her HF and to gain in his/her own confidence, another said to feel supported already by the nurse’ visit. One person reported to feel neutral about the intervention and described his/her experience of monitoring symptoms as tedious but that the activity of symptom monitoring also helped to interpret symptom increase and to be aware of the health situation. A person with cognitive impairment felt absolutely healthy and not concerned with HF. Some persons commented their TAP answers related to less intervention acceptability: one person reported not wanting to participate again because of the burden to participate related with impaired vision and audition, one reported the intervention duplicating the follow-up already done by the general practitioner and the fact that death will concern everyone, finally the person who discontinued the intervention wrote not being interested in the interactions because it repeated what the person already knew.

Two informal caregivers commented their TAP answers, one acknowledging for the intervention and one other mentioning being happy for the participation but not wanting to participate again related to the burden of participation of her mother with impaired vision and audition.

Three nurses commented their TAP answers, one questioning what the persons with HF remembered after the intervention related with their impaired cognition. Another mentioned her frustration to have delivered the intervention to only three persons with HF. She reported the presence of GCS with her at home when delivering the intervention as beneficial in supporting to deliver the intervention and would recommend it at the first interaction. A nurse mentioned the difficulty to conciliate her part-time job of home-care nurse and the intervention delivery. She reported her participation as being enriching.
